# 

**Published:** 1856-07

**Authors:** 


### SUBSCRIPTIONS RECEIVED.

Jas. H. Price, Ga.; H. L. Byrd, Ga.; Dr. —— Coe, Ga.; W. A. Culbertson, Ga.; C. B. Slaughter, Ala.; Wm. M. McLean, Ga.; N. H. Baker, Ala.: H. J. Sanders, Ala.; Charles Elliott, Ala.; S. E. Bell, Ala.; J. G. Broils, Ala.; E. C. Moyer, Ga.; A. P. Cannon, Ga.

### TO CORRESPONDENTS.

☞ All Communications pertaining to the Editorial Department of the Journal, should be addressed to the Editors.

☞ All Communications, having reference to the Subscription, the Advertising or the Financial Department, should be addressed to the Treasurer, J. G. Westmoreland, M. D., Dean of the Faculty of the Atlanta Medical College.

---

☞ Messrs. A. Tilden and W. H. Phillpot are authorized Agents for this Journal.

